# Preparations in Scotland

**Published:** 1914-08-29

**Authors:** 


### Preparations in Scotland.

### ABERDEEN, GLASGOW, AND PERTH.

*(From our Special Correspondents.)*

Dr. William Sinclair, medical superintendent of the Royal Infirmary, Aberdeen, has left for service with the Territorial Forces, and Dr. John Scott Riddell, M.V.O., is acting in his stead.

The offer by the directors of the Aberdeen Royal Infirmary of 150 beds has been accepted by the Admiralty. A number of the members of the honorary staff have been called up, but arrangements have been made for the temporary staffing of the hospital. Five of the six resident medical officers have volunteered for active service, but their places have been satisfactorily filled. The acting medical superintendent, Mr. J. Scott Riddell, M.V.O., senior surgeon to the hospital, and as explained above, now in charge, has organised a complete service for the transport of the wounded from the harbour or railway station, thanks to the voluntary offer of over fifty motor-cars and lorries. The hospital is utilised for naval casualties; about forty-five medical students have volunteered to act as dressers. Transport assistance will also be forthcoming from the station railway squad and from the Boys' Brigade.

The managers of the Glasgow Royal Infirmary have offered, should need arise, accommodation for sick and wounded. They are in a peculiarly favourable position for affording accommodation at this time, seeing that, as the new infirmary has just been opened and as certain parts of the old buildings still remain, at least 100 beds are immediately available. In addition to this, further accommodation would be afforded if necessary.

The old Perth Infirmary was vacated last month, when the patients were moved to the new building. It has been used up to last Saturday for billeting soldiers. These have since left Perth, and, if the old building is not further required for billeting purposes, the directors have resolved to offer it to the War Office as a base hospital.

Into the new infirmary cases of serious illness or accident among the troops will be received, and with this object in view arrangements are being made as far as possible to discharge convalescent patients so as to make room for emergency or more urgent cases.

No. 3 and No. 4 General Hospitals at Stobhill have been placed at the disposal of the Territorial Force Association by the Glasgow Parish Council. The several years' old arrangement between the Parish Council and the Association came into force automatically on the outbreak of war. The institutions are understood to be fully equipped and ready. The usual equipment has been carried out by the Association, and all auxiliary help is being provided by the Scottish branch of the British Red Cross Society. A service of motor cars has been arranged for, which will be ready on the requisition of officers commanding the hospitals. In the meantime, the Parish Council have removed 800 patients to other institutions belonging to them. Thus the Territorial Association have accommodation for 1,040 patients, which can be increased if required.

The offer of the Falkirk Infirmary to equip the Victoria School, Falkirk, as a hospital, has been acknowledged by the Director of Medical Services (Scottish Command), but not yet accepted.

### KILMARNOCK.

*(From our Special Correspondent.)*

The directors of Kilmarnock Infirmary, an institution with one hundred beds, are prepared to provide twenty-five beds, if requested, for the sick and wounded. Members of the Red Cross Society, to the number of twelve, are offered daily training in the hospital. Ladies' work parties are being formed in several districts to provide clothing, and meetings have been held in support of the National Relief Fund. Halls are also being selected for the treatment of the sick and wounded.

### STIRLING.

In Stirlingshire the Red Cross Society, of which Mr. James A. Gibson is honorary secretary, is being very active. At the Stirling Royal Infirmary the directors have placed the male ward at the disposal of the Duke of Montrose, as President of the Stirlingshire Territorial Forces Association. This gives twenty-four beds, including medical, surgical, and nursing treatment. Several individuals have placed their private houses at the disposal of the Red Cross Society for hospital accommodation.

### THE SCOTTISH RED CROSS SOCIETY.

The Scottish Red Cross Society, working in conjunction with the naval and military authorities, is in a position to state that ample hospital accommodation is at present available in Scotland for any immediate demands likely to be made upon it. The military authorities have given instructions that no steps are to be taken to fit out any additional hospital until definite orders have been received to do so by the Scottish Red Cross Society, the headquarters of which are at 137 Sauchiehall Street, Glasgow. The information we have received is that the military authorities have arranged for 1,040 hospital beds to be ready in Glasgow, 520 hospital beds at Edinburgh, and a similar number at Aberdeen. The Cromarty Naval Hospital contains 400 beds. The British Red Cross Society is in charge of the organisation of the voluntary workers, chiefly women, who are willing to make garments and provide various appliances likely to be useful.

The St. Andrew's Ambulance Association has taken over all the regular military hospitals in Scotland under an arrangement made during peace time with the War Office. The staffing of these hospitals with medical men, nurses, and orderlies is also in the hands of this Association, which has set free the Army officers for military service. The St. Andrew's Association is also providing a large amount of ambulance material, including stretchers. It may be well to explain that this Association fulfils in Scotland the same functions as those of the St. John Ambulance Association in England. The St. Andrew's Ambulance Association has issued an appeal for articles of clothing, gifts, and money.

